# Chronic hyperglycemia and intracranial meningiomas

**DOI:** 10.1186/s12885-024-12243-4

**Published:** 2024-04-17

**Authors:** D. Orešković, A. Madero Pohlen, I. Cvitković, J.F. Alen, M. Raguž, A. Álvarez-Sala de la Cuadra, G.J. Bazarra Castro, Z. Bušić, I. Konstantinović, V. Ledenko, C. Martínez Macho, D. Müller, M. Žarak, N. Jovanov-Milosevic, D. Chudy, T. Marinović

**Affiliations:** 1 Department of Neurosurgery, Clinical Hospital Dubrava, Zagreb, Croatia; 2 Department of Neurosurgery, University Hospital de la Princesa, Madrid, Spain; 3 Department of Neurosurgery, University Hospital Center Split, Split, Croatia; 4 Department of Pathology, Clinical Hospital Dubrava, Zagreb, Croatia; 5 Clinical Department of Laboratory Diagnostics, Clinical Hospital Dubrava, Zagreb, Croatia; 6 Faculty of Pharmacy and Biochemistry, University of Zagreb, Zagreb, Croatia; 7 Department of Biology, School of Medicine, University of Zagreb, Zagreb, Croatia; 8 Scientific Centre of Excellence for Basic, Clinical and Translational Neuroscience, School of Medicine, Croatian Institute for Brain Research, University of Zagreb, Zagreb, Croatia; 9 Department of Neurology and Neurosurgery, Faculty of Dental Medicine and Health, Josip Juraj Strossmayer University of Osijek, Osijek, Croatia

**Keywords:** Meningioma, Hyperglycaemia, Dexamethasone

## Abstract

Meningiomas are among the most common primary tumors of the central nervous system. Previous research into the meningioma histological appearance, genetic markers, transcriptome and epigenetic landscape has revealed that benign meningiomas significantly differ in their glucose metabolism compared to aggressive lesions. However, a correlation between the systemic glucose metabolism and the metabolism of the tumor hasn’t yet been found. We hypothesized that chronic levels of glycaemia (approximated with glycated hemoglobin (HbA1c)) are different in patients with aggressive and benign meningiomas. The study encompassed 71 patients with *de novo* intracranial meningiomas, operated on in three European hospitals, two in Croatia and one in Spain. Our results show that patients with WHO grade 2 meningiomas had significantly higher HbA1c values compared to patients with grade 1 lesions (*P* = 0.0290). We also found a significant number of patients (19/71; 26.7%) being hyperglycemic, harboring all the risks that such a condition entails. Finally, we found a significant correlation between our patients’ age and their preoperative HbA1c levels (*P* = 0.0008, ρ(rho) = 0.388), suggesting that older meningioma patients are at a higher risk of having their glycaemia severely dysregulated. These findings are especially important considering the current routine and wide-spread use of corticosteroids as anti-edematous treatment. Further research in this area could lead to better understanding of meningiomas and have immediate clinical impact.

**Supplementary Information:**

The online version contains supplementary material available at 10.1186/s12885-024-12243-4.

## Introduction

Meningiomas are among the most common tumors of the central nervous system (CNS), accounting for roughly 30% of all primary CNS neoplasms [1]. They are present in around 1% of the general population, exhibiting an incidence of approximately 7.8 / 100 000 and a prevalence of about 97 / 100 000. Meningiomas originate from arachnoid cap cells, are usually estimated to grow 2–4 mm/year and are slightly more common in females [2–4]. Several risk factors for meningioma development have been investigated, with age, radiation and sex hormones being most commonly reported as elevating the risk of the disease [5, 6], while smoking, hypertension and the metabolic syndrome still being mostly inconclusive as risk factors [2–4]. According to the current WHO classification [7], roughly 80% of meningiomas are considered grade 1 lesions, with a 10-year overall survival rate of up to 90%. WHO grade 2 tumors constitute around 20% of all meningiomas and have a 10-year overall survival rate of about 53%. Meningiomas which are diagnosed as WHO grade 3 are the least frequent, being found in only 2–3% of patients and having by far the worst prognosis with a 10-year overall survival rate of 0% [2–4]. In addition to the characteristics of the tumor itself, there is a growing awareness that several other external factors can also influence the survival of meningioma patients [8]. Complete surgical excision remains the primary treatment modality for all types of meningiomas, with radiation therapy being a supplementary treatment option. There are no chemotherapeutic agents currently in routine use for the treatment of patients with meningiomas [9–11].

Due to more diagnostics being available worldwide, more and more meningiomas are being found incidentally. Optimal management of these lesions is still unclear [12, 13]. It is also becoming increasingly evident that histopathology and DNA mutations do not fully capture the vast biological and clinical heterogeneity of these tumors [14–16]. Meningioma classification has, therefore, emerged as one of the most important areas of research in recent years [17].

While metabolic activities in healthy cells rely primarily on mitochondrial oxidative phosphorylation in order to generate adenosine triphosphate (ATP), in tumor cells this mechanism is altered. Indeed, most cancer cells use glycolysis as a means of energy production, while oxidative phosphorylation is significantly reduced. This means that proliferating malignant tissues extract energy from glucose in a very inefficient manner, making them highly susceptible to glucose deprivation. Such an active metabolic change to a less efficient glucose metabolism has been known ever since the pioneering work of Otto Warburg [18, 19], and is today considered one of the hallmarks of cancer [20, 21]. The hypothesized reasons for such an unintuitive phenomenon are beyond the scope of this article and are discussed at length elsewhere [22].

Meningiomas also exhibit this peculiar behavior of neoplastic cells. For example, visualizing the metabolism of a radioactive glucose analogue, fluorodeoxyglucose (^18^F-FDG) on PET, can predict both the meningioma proliferation and its grade [23–28]. Similar results have been shown when investigating the meningioma gene expression [29], transcriptome and proteome [30], DNA methylation patterns [31, 32] and metabolomics [33–35]. All of this data seems to suggest that, in accordance with Warburg’s findings over a century ago, meningioma aggressiveness is significantly correlated to their metabolism, which may allow for early detection of clinically aggressive tumors despite their benign histological appearance.

Considering the importance of glucose in tumor cellular biology, much effort has been made trying to elucidate the relationship between systemic glucose metabolism and the development of a malignant disease. And, as mentioned earlier, while being a significant risk factor for cancer in general [36–39], the role of glycaemia, diabetes, metabolic syndrome and obesity as risk factors for meningiomas isn’t as straightforward. Indeed, the literature on the topic remains remarkably divided [40, 41]. However, another aspect of the connection between systemic glucose metabolism and the biology of a meningioma is the influence which glycaemia could exert on a formed tumor. Also, while it is known that elevated glycemia is prevalent in the general population, especially in elderly people, it is also known that hyperglycemic elderly people also constitute a large number of meningioma patients. The research into this possible connection is currently lacking. Our research therefore aimed to determine whether chronically hyperglycemic patients are more likely to be diagnosed with a higher-grade meningioma and to determine the possible interconnectedness between the patients’ glycated hemoglobin (HbA1c), BMI, age and the Ki67 reactivity of the tumor, as a marker of cellular proliferation.

## Materials and methods

### Patient selection

This analysis was conducted on patients who underwent either a complete resection or a subtotal reduction of their intracranial meningioma at three institutions, the (i) Department of Neurosurgery, Clinical Hospital Dubrava, Zagreb, Croatia, (ii) Department of Neurosurgery, University Hospital Center Split, Split, Croatia and (iii) Department of Neurosurgery, University Hospital de la Princesa, Madrid, Spain. HbA1c measurement was added to the routine preoperative analysis of patients with an intracranial tumor in all three institutions. All patients reported here were operated on and diagnosed after implementing the new WHO grading scheme in 2021. Informed consent was obtained from each patient before collecting blood samples in all three institutions, and this analysis was conducted with the approval of the Ethical Committee of each hospital, with accordance to the Declaration of Helsinki and with accordance to all of the relevant national legislature in each respective country.

Several exclusion criteria were put in place in order to homogenize our cohort. Patients whose tumor mass was predominately (> 90%) in the intracranial space were included in the study, otherwise they were excluded. Considering how spinal meningiomas seem to be different clinical entities compared to intracranial meningiomas, especially concerning their cellular proliferation [42], patients with spinal meningiomas were all excluded from the study. Patients who underwent any type of blood transfusions or had a significant surgical treatment in the 3 months prior to their hospitalization were excluded since this rendered their HbA1c levels unreliable. Several patients were excluded on the basis of the fact that they had recurring meningiomas, having already previously undergone surgical resection of the tumor with or without subsequent radiotherapy, which has dramatically changed the tumor itself and its cellular biology, making them incomparable to the meningioma cells of patients with *de novo* lesions. Patients with inconclusive pathohistological results were all excluded from the study. Patients whose HbA1c levels were taken postoperatively due to technical reasons, were also excluded from the analysis. Patients who were harboring other malignant diseases at the time of surgery, both of the central nervous system and/or other sites, were all excluded from the study. In total, there were 71 patients included in our study who were diagnosed with a WHO grade 1 or a WHO grade 2 meningioma (Table 2 - Supplement).

### HbA1c measurement and dexamethasone

HbA1c measurement was added to the routine preoperative blood testing in all patients who were admitted to the neurosurgery departments of the aforementioned three institutions for surgery of an intracranial neoplasm. All the blood samples were taken up to three days after the patients’ admission to the hospital and up to four days before surgery. The patients’ blood samples were drawn and analyzed in a routine manner, with commercially available measuring instruments which analyze HbA1c using the current gold-standard method, a high-performance liquid chromatography (HbA1c is calculated as a ratio to total hemoglobin using a chromatogram). The testing was performed on the same day that the blood sample was obtained.

All of the patients involved in this study were receiving corticosteroid treatment when their HbA1c was measured (Dexamethasone 2 × 8 mg iv./day). They were receiving the therapy for up to three days before their blood samples were taken. If they were receiving Dexamethasone for a longer period of time, they were excluded from the study.

### Pathohistological analysis

The tumor tissue was processed using standard histological methods. The tissue was fixed in 10% formalin solution, (neutral buffered, Sigma-Aldrich, MO, USA), then embedded in paraffin, tissue blocks, sliced to the thickness of 5 mm, then stained with H&E and finally analyzed using a microscope.

Proliferation factor Ki67 was analyzed immunohistochemically in all three institutions, according to the current standards and in accordance with the antibody manufacturer’s specifications. At least 1,000 nuclei were counted at high magnification (40× objective) without recounting the same areas and the average was expressed as the percentage. Foci of necrosis were excluded. Due to the fact that meningiomas are not always homogeneous neoplasms with regard to their cellular proliferation (there can be different loci of proliferation inside the same tumor), the pathologist’s report can often state different values of Ki67. If this was the case, the highest reported Ki67 value was included in the study. Also, beside the pathologist who examined the sections and stated the diagnoses, another pathologist verified the results.

### Statistical analysis

Statistical analysis was performed using MedCalc statistical software, version 19.1.1 (MedCalc 22.014, Mariakerke, Belgium). The level of significance was set at *p* < 0.05. Normality testing with Kolmogorov-Smirnov test showed rejection of normality distribution, so nonparametric tests were used. All numerical variables were presented as median and interquartile range (IQR).

A Mann–Whitney U test assessed the statistically significant difference in HbA1c (%) values between the meningioma WHO grades 1 and 2 patients. Spearman’s correlation coefficient (rho, ρ) was used to determine the correlation between HbA1c and patients’ age, BMI and Ki67 expression. A dot-plot (all data shown) was used for graphic data comparison.

## Results

### Description

The study included 71 patients aged from 36 to 86 years (median age 63 years) with a confirmed diagnosis of an intracranial meningioma (Table 2 - Supplement). The majority of subjects were female (*N* = 55; 77%). WHO grade 1 meningioma was pathohistologically confirmed in 53 patients (74.6%), and 18 patients (25.3%) were diagnosed with a WHO grade 2 meningioma. The overall measured values of HbA1c ranged from 5.1 to 11.2%.

**Fig. 1:**
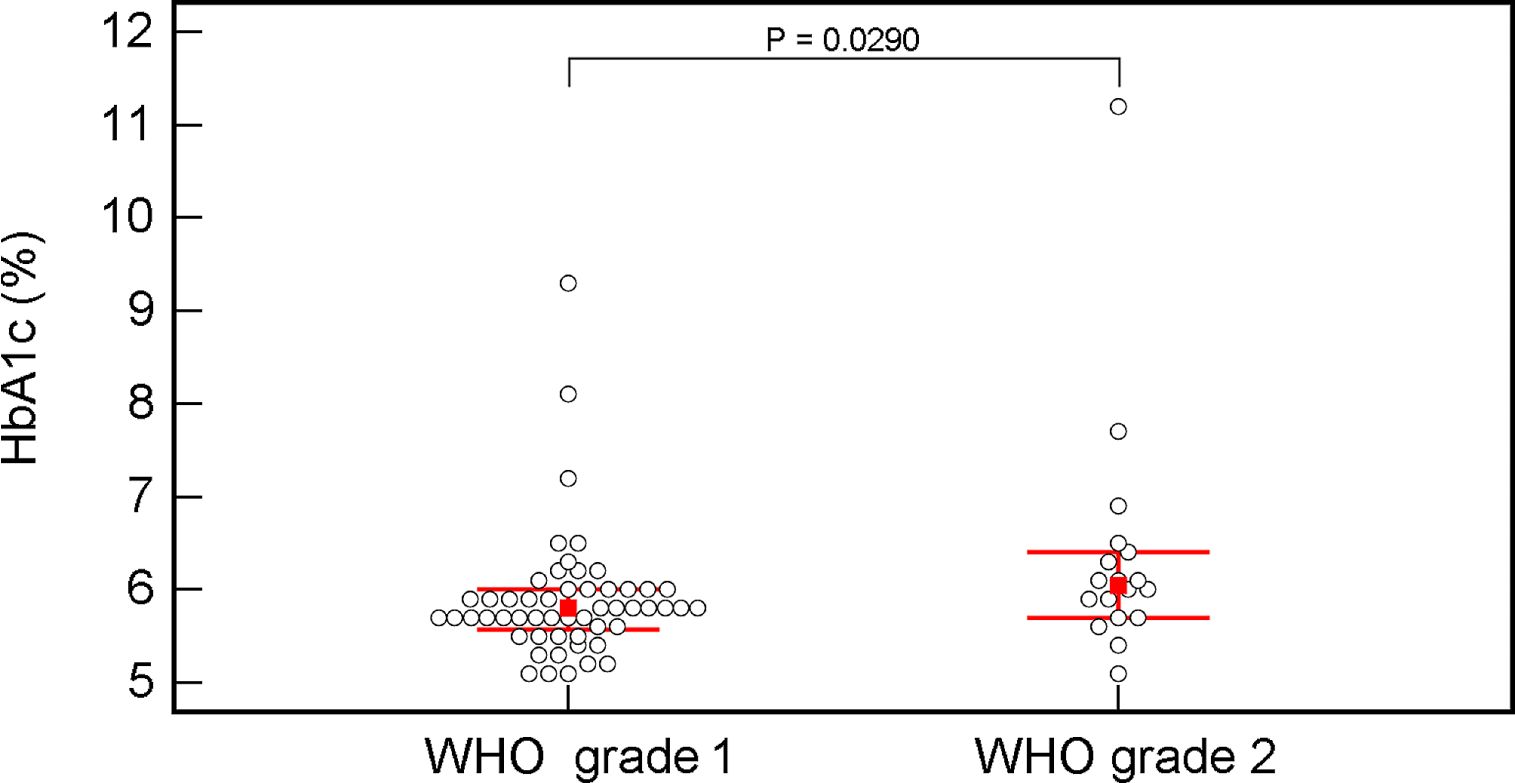
Dot-plot (all data presented) showing differences in HbA1c (%) values between patients with WHO grade 1 and WHO grade 2 meningiomas. Empty circles are individual HbA1c values, the red squares represent the median, and the horizontal red lines represent the interquartile range (IQR)

Patients with WHO grade 2 meningioma had significantly higher HbA1c values than patients with WHO grade 1 meningioma (*P* = 0.0290) (Table 1; Fig. 1). Grade 2 meningiomas also exhibited higher levels of Ki67 (Table 1). There was no statistically significant difference in BMI (*P* = 0.7361) or age (*P* = 0.7862) between patients with different tumor grades (Table 1). Nineteen patients (19/71; 26.7%) had HbA1c value higher than 6.0%. Of these, twelve were previously undiagnosed with DM and were not receiving any anti-diabetic medication.

**Table 1:** General patient characteristics with data presentation and statistical analysis depending on WHO grades of a meningioma. Note the statistically significant difference in the patients’ HbA1c between patients with different meningioma grades. Note also that grade 2 meningiomas had a higher expression of Ki67 reactivity, indicating that they exhibited more cellular proliferation

Characteristics	All patients (*N* = 71)	WHO grade 1 (*N* = 53)	WHO grade 2 (*N* = 18)	P (Mann-Whitney)
1. Age*	63 (36-84)	63 63 (36-84)	64 (48-80)	0.7862
2. BMI**	27.4 (24.6–30.0)	28.2 (24.6–30.1)	27.0 (24.6–29.7)	0.7361
**Variables**				
1. HbA1c (%)**	5.8 (5.6–6.1)	5.8 (5.6–6.0)	6.1 (5.7–6.4)	**0.0290**
2. Ki67 (%)**	6.0 (3.0–14.8)	5.0 (2.0–10.5)	13.5 (7.0–20.0)	**0.0001**

BMI– body mass index; * median and whole range; ** median and interquartile range

**Fig. 2:**
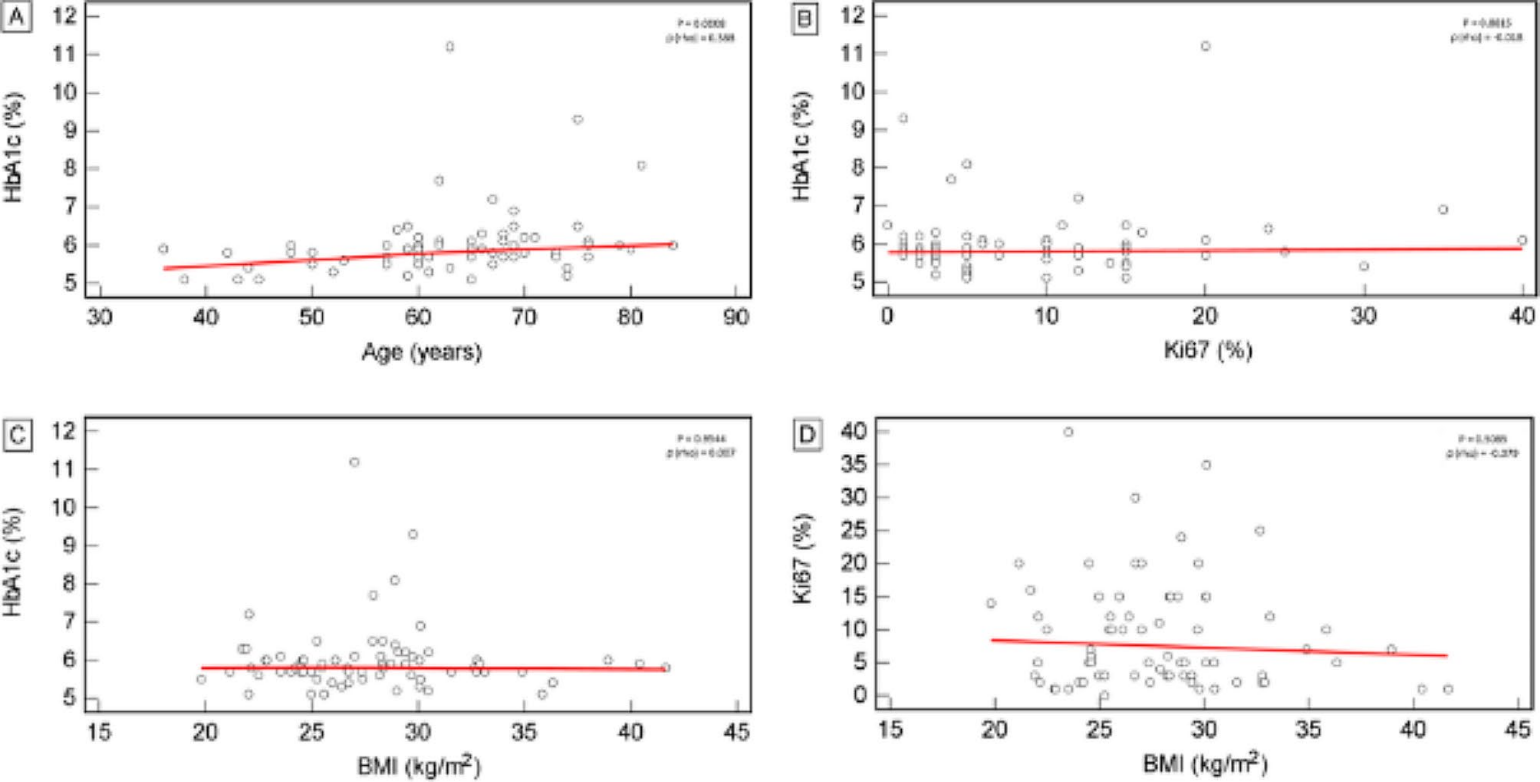
Correlation between patients’ characteristics (age, BMI) and measured variables (HbA1c, Ki67). **A**– HbA1c and age correlation, **B**– HbA1c and Ki67 correlation, **C**– HbA1c and BMI correlation, **D**– BMI and Ki67 correlation. Red line in each figure represents a regression line

We found statistically significant positive correlation between the patients’ age and their HbA1c values (*P* = 0.0008, ρ(rho) = 0.388) (Fig. 2A). Contrary to our preliminary research [43], in the current study there was no significant correlation between the patients’ HbA1c and Ki67 values (*P* = 0.8815, ρ(rho)=-0.018) (Fig. 2B). Finally, there was no significant correlation between the patients’ BMI and their HbA1c (*P* = 0.9544, ρ(rho) = 0.007) (Fig. 2C) or their Ki67 expression (*P* = 0.5085, ρ(rho)=-0.079) (Fig. 2D).

## Discussion

### Glycaemia and meningioma aggressiveness

The aim of this study was to determine whether chronically hyperglycemic patients were more likely to harbor grade 2 meningioma compared to normo-glycemic patients. The found differences in HbA1c levels which we observed, could primarily be explained through the influence of the available blood glucose on meningioma biology. Indeed, it is known that chronically elevated glycaemia causes a variety of effects which could in turn be connected to higher proliferation and aggressiveness of a meningioma. This is possible through (i) the advanced glycation end products (AGEs), (ii) DNA methylation and (iii) meningioma hormonal receptors.

(i) AGEs are normal proteins or lipids that become glycated after contact with sugars, with more glycation occurring during prolonged exposure. One prominent example is the glycation of hemoglobin, which correlates well with the average glycaemia levels over the life span of erythrocytes, making it a reliable marker of chronic glycaemia [44]. This process can occur with many more proteins and lipids in a hyperglycemic milieu, contributing to the development of atherosclerosis and various micro- and macrovascular complications [45, 46]. Furthermore, the presence and accumulation of AGEs in different cell types are known to significantly alter both the intracellular as well as the extracellular structure and function, primarily influencing specific genes and transcription factors, and ultimately leading to diabetes and cancer [47]. Indeed, the presence of AGEs is known to correlate significantly with the development of various neoplasms such as gallbladder [48], hepatocellular, prostate and breast cancer [49]. Similar findings have been confirmed in meningiomas as well, where glycation was shown to cause changes in the expression of enzymes with a known role in tumor progression [50] as well as an increase in the invasion of a meningioma [51].
(ii) The epigenome is recognized as an important factor in the pathophysiology of many diseases, including cancer [52]. While several external factors affect the degree and pattern of DNA methylation, arguably one of the most important ones is glycaemia. Indeed, the effects of hyperglycemia on DNA methylation are already well-known and have been shown in various tissues [53–56]. These effects depend on the exact DNA locus [57] and can last up to several years [52, 58]. In fact, one of the mechanisms of action of current antidiabetic medication is precisely by changing the DNA methylation patterns [59]. Of note is that in addition to DNA, methylation occurs on RNA and protein levels as well, which is rapidly emerging as a novel target in cancer [60, 61]. Such findings seem to hold true for meningiomas as well. Indeed, meningioma DNA methylation is already considered an important biomarker [31, 62]. For example, the FOXM1 gene, a mitotic transcription factor implicated in a variety of malignant diseases and particularly enriched in invasive meningiomas, is known to be tightly controlled by the epigenetic landscape, with aggressive meningiomas being characterized by DNA hypermethylation [32, 63]. This hypermethylation could thus, at least in part, be perpetuated by chronic hyperglycemia.
(iii) Hormones which are heavily involved in systemic glucose metabolism and glycolysis, such as the insulin-like growth factor (IGF), have already been shown to be a reliable marker of aggressive meningiomas [29]. Indeed, sporadic reports have already suggested that inhibition of the IGF receptor (IGF-1R) could have a role in future meningioma treatment [64]. Moreover, the expression of IGF-1R and its’ impact on cellular proliferation have been shown to be significantly influenced by glycaemia levels in other malignancies [65, 66], indicating that a similar process could be possible in meningiomas as well. Somatostatin is another hormone that is heavily regulated by glucose concentrations [67]. One type of receptor for this hormone, the somatostatin receptor 2 (SSTR2), is expressed in virtually all meningioma cells, with the exact importance of this finding still being unknown [23, 68]. It has, however, been repeatedly shown that a PET radiotracer which binds to the receptor ([^68^Ga]Ga-DOTA-SSTR) can reliably predict meningioma growth, grade and recurrence [23, 24, 27], arguably pointing to the fact that meningioma biology is heavily dependent on the metabolism of glucose.

In summary, considering the well-known effects which occur during hyperglycemia in both healthy and malignant tissues, it seems likely that similar changes could also happen in meningiomas. However, larger in vitro and in vivo studies are needed to determine the exact effects which hyperglycemia could exert on meningioma biology and clinical behavior.

### Hyperglycemia

Another important finding of our research is a large percentage of patients being hyperglycemic (having HbA1c > 6.0). This is important since hyperglycemia, especially during prolonged periods, is known to cause a variety of multi-systemic pathologies which include atherosclerosis, neuropathy, nephropathy and retinopathy [45, 69]. Indeed, hyperglycemia has been associated with an increased risk of perioperative complications, higher morbidity, and a 10% of all-cause mortality of the world population [69–71]. Moreover, hyperglycemia is a factor most commonly associated with adverse effects during oncological treatment. These effects include a higher risk of infections, toxicity, morbidity, chemo-resistance, pain, fatigue, depression and sleep disturbance, as well as a decrease in overall and disease-free survival with frequent recurrence, progression and metastasis of the malignant disease [72, 73]. However in meningioma patients, this type of research has been remarkably scarce.

Global surveys of diabetes prevalence estimate that around 8% of the world population aged 18–99 live with diabetes, with half of them being undiagnosed. Another 7% of the population is thought to have some impairment in their glucose tolerance [74]. Our results show 26.7% (19/71) of patients with intracranial meningiomas having elevated HbA1c. In renal transplant patients, for example, it is recommended that if their HbA1c levels are equal to or higher than 5.8%, they should undergo further diagnostics in order to determine if they have diabetes [75]. Compared to our results, we found that 57.7% (41/71) of our patient cohort would satisfy these criteria and would warrant further evaluation. Such guidelines and diagnostic criteria do not currently exist for meningioma patients and are difficult to interpret between patients with different diseases, but they still give a general idea of the problem at hand. Moreover, nine of our patients (9/71, 12.6%) had HbA1c values higher than or equal to 6.5%. Such high values immediately indicate diabetes mellitus without further diagnostics [76]. Of these patients, three of them were undiagnosed with diabetes before hospitalization. In other words, 4.2% (3/71) of our entire patient cohort was harboring severe undiagnosed and untreated hyperglycemia and could be diagnosed with diabetes without any further diagnostics. Of note, 15.5% (11/71) of our patients were previously diagnosed with diabetes (Table 2– Supplement). And most of these previously diabetic patients still had severe glycaemia dysregulation despite their routine anti-diabetic medication and regular diabetologists’ consultations.

It is also well-known that elderly patients are at a higher risk of suffering from deleterious consequences of hyperglycemia, such as a life-threatening hyperosmolar state which requires aggressive hydration and insulin therapy [77]. This is in accordance with our results, since we found a significant correlation between our patients’ age and their preoperative HbA1c levels (*P* = 0.0008, ρ(rho) = 0.388). In other words, our older patients were at a higher risk of having their glycaemia severely dysregulated. Such findings would in turn imply that glycaemia of elderly meningioma patients should be more closely monitored, with their preoperative diabetic status examined more rigorously.

### Dexamethasone

Oncological patients are often susceptible to iatrogenic glycaemia dysregulation, occurring primarily through corticosteroid therapy. It is estimated that a large percentage of patients world-wide (up to 10%) are currently being prescribed corticosteroid treatment, including those with cancer [78]. Such a large number is thought to rise in the future [77]. This is true for brain tumor patients as well, as corticosteroid therapy with intravenous Dexamethasone in a dose of 2 × 8 mg/day has been standard treatment against peritumoral brain edema for over 60 years [79]. And the tendency of corticosteroids to induce hyperglycemia is one of the most well-known consequences of Dexamethasone treatment. Indeed, it is estimated that around 30% of patients being prescribed glucocorticoids are hyperglycemic, whereas around 18% of them develop SIDM (Steroid-Induced Diabetes Mellitus) [76]. In fact, the magnitude of the problem is actually thought to be underestimated because corticosteroids usually cause post-prandial hyperglycemia which is seldom found by looking at fasting blood glucose [76, 77].

Due to the high prevalence and severity of the issue, there have been multiple attempts to standardize the diagnostics and treatment of patients suffering from SIDM [76, 78, 80–82]. As mentioned earlier, guidelines have been published for patients receiving corticosteroids during chemotherapy [83] or after renal transplantation [75]. Based mostly on best clinical practice and not rigorous scientific studies [78], these guidelines nevertheless provide valuable clinical information. Such protocols are unfortunately missing in neuro-oncology, and glycaemia and the possibility of SIDM is rarely, if ever, mentioned in the current guidelines on the management of meningioma patients [84]. In our research, we attempted to minimize the effects of corticosteroids on HbA1c by analyzing it in the first 3 days of Dexamethasone treatment, since measuring HbA1c before any such treatment has proven challenging from a technical perspective (see Limitations below).

Our study is arguably the first attempt to estimate the number of chronically hyperglycemic meningioma patients. Knowing the high prevalence and incidence of meningiomas, the fact that such a large number of patients harboring this disease could have severe, undiagnosed and untreated hyperglycemia is indeed a cause for concern. Our results suggest that all meningioma patients, especially the elderly with a previous diagnosis of diabetes, should have their diabetic status routinely examined preoperatively. This should be performed not just because of the possible connection of glycaemia and tumor proliferation, but also because of the many potentially severe complications occurring due to unrecognized hyperglycemia during peri- and post-operative treatment [85]. Notably, hyperglycemia in brain tumor patients could also be important in the context of venous thromboembolism [86]. Also, of note is that we only investigated patients who underwent open surgery. A large number of meningiomas are only incidentally found and don’t undergo surgery, with glycaemia levels of these patients remaining unknown.

## Limitations

The main limitation of our study is a modest number of participants tested. This has made it impossible to test whether or not other factors could be connected to different levels of glycaemia, such as the tumor location or the exact meningioma subtype. However, we feel that our results, beside the fact that HbA1c measurement is both routine and widely available in many institutions, should encourage other researchers to implement the measurement in their routine preoperative assessment and to publish their results. We feel that the number of patients we tested is sufficient to warrant interest into this currently overlooked area of neuro-oncology. Secondly, no patients in our analysis were diagnosed with grade 3 meningiomas. Being by far the least frequent, we couldn’t analyze patients with these tumors and their possible relationship with systemic glucose metabolism remains unknown. Thirdly, no causal relationship which glycaemia and meningiomas could have with each other was proven. However, the fact that patients with grade 2 meningiomas could have significantly higher levels of HbA1c, is something which, to the best of our knowledge, has not been shown so far.

HbA1c is today considered a robust and reliable marker of chronic glycaemia. Indeed, it is one of the cornerstones of modern treatment of metabolic diseases. However, HbA1c measurement can also be influenced by several factors other than glycaemia levels [87]. The most common ones which lead to unreliable and misinterpreted values of HbA1c include ethnic differences, various conditions which affect the erythrocyte life span or glycation of hemoglobin, iron and/or vitamin B12 deficiency, several types of diseases (coronary, liver, spleen). Age and obesity are also known to influence the HbA1c values, and so are various medications (Aspirin, opioids, antivirals, hydroxyurea,… [88]). In our research we could have disregarded most of these limitations. For example, our cohort consisted only of Caucasian population in Croatia and Spain. It also consisted of relatively healthy individuals notwithstanding their meningioma, since any of the aforementioned conditions and diseases would make the patient unfit for elective surgery until the condition is resolved. However, researchers trying to replicate our results should definitely keep in mind the limitations of HbA1c measurement.

As mentioned earlier, all of our patients received corticosteroid treatment during few days before measurement (three at most), and HbA1c was measured a few days before surgery (usually three or four). This leaves a slight discrepancy between the HbA1c measurement and the pathohystologic analysis of the tumor tissue, as well as a possibility that severe iatrogenic glycaemia dysregulation could occur during this short period of time. Indeed, it has been recognized that even small doses of Dexamethasone can have a significant effect on glycaemia of neurosurgical patients [89–91]. However, the effects which such short-term Dexamethasone treatment could exert on HbA1c and chronic glycaemia levels in these patients remain unknown. Even if this is the case however, we are convinced that our conclusions still hold true, and that a significant percentage of meningioma patients are at a high risk of hyperglycemia, either spontaneously, or through iatrogenic intervention. Thus, considering how HbA1c is such a ubiquitous and reliable measurement, we are confident that our results are indicative of these patients chronic, preoperative glycaemia. Future research will however have to consider both the number of patients who are hyperglycemic before any corticosteroid treatment, and the number of hyperglycemic patients during and after such therapy.

## Conclusions

It is becoming increasingly apparent that in order to fully encapsulate the vast heterogeneity of meningiomas, future research will have to focus not just on their histological appearance and genetic markers, but also their epigenetic landscape, proteome and transcriptome and a myriad of other external variables. Based on our results, we propose that systemic glucose metabolism is also an important factor in meningioma development and progression, and is currently being overlooked. Our results also suggest that patients with intracranial meningiomas could be at a high risk of suffering from unrecognized and untreated glycaemia dysregulation with all the many risks that such a condition entails. This is especially important considering the current routine and wide-spread use of corticosteroids as anti-edematous treatment. The well-known effects of hyperglycemia on a wide range of tissues and organs suggest that a similar mechanism could be found in meningiomas as well, and possibly mediated during treatment of patients with these common tumors.

### Electronic supplementary material

Below is the link to the electronic supplementary material.

Supplementary Material 1

## Data Availability

All data presented in the study are available from the corresponding author upon reasonable request.

## Abbreviations

CNS: Central Nervous System
HbA1c: Glycated Hemoglobin
BMI: Body Mass Index
ATP: Adenosine Triphosphate
^18^F-FDG: Fluorodeoxyglucose
AGE: Advanced Glycation End products
IGF: Insulin-like Growth Factor
SIDM: Steroid-Induced Diabetes Mellitus
